# A master of nursing science curriculum revision for the 21st century – a progress report

**DOI:** 10.1186/s12909-019-1588-9

**Published:** 2019-05-08

**Authors:** René Schwendimann, Katharina Fierz, Elisabeth Spichiger, Brenda Marcus, Sabina De Geest

**Affiliations:** 1 Departement Public Health DPH, Nursing Science, Faculty of Medicine, University of Basel, Bernoullistrasse 28, 4056 Basel, Switzerland; 2 Patient Safety Office, University Hospital Basel, Spitalstrasse 22, 4031 Basel, Switzerland; 3 Head Office of Nursing and Allied Health Professionals, Inselspital Bern University Hospital, Freiburgstrasse 44a, 3010 Bern, Switzerland

**Keywords:** Curriculum, Nursing education, Master of science in nursing, ANP

## Abstract

**Background:**

Preparing a 21^st^ century nursing workforce demands future-oriented curricula that address the population’s evolving health care needs. With their advanced clinical skill sets and broad scope of practice, Advanced Practice Nurses strengthen healthcare systems by providing expert care, especially to people who are older and/or have chronic diseases. Bearing this in mind, we revised our established Master of Nursing Science curriculum at the University of Basel, Switzerland.

**Methods:**

Guided by the Advanced Nursing Practice framework, interprofessional guidelines, fundamental reports on the future of health care and the Bologna declaration, the reform process included three interrelated phases: *preparation* (work packages (WPs): curriculum analysis, alumni survey), *revision* (WPs: program accreditation, learning outcomes), and *regulations* (WPs: legal requirements, program launch).

**Results:**

The redesigned MScN curriculum offers two specializations: ANP and research. It was implemented in the 2014 fall semester.

**Conclusions:**

This curriculum reform’s strategic approach and step-by-step processes demonstrate how, beginning with a solid conceptual basis, congruent logical steps allowed development of a program that prepares nurses for new professional roles within innovative models of care.

## Background

Population health is inseparable from care provision. Epidemiological and demographic shifts (towards chronic disease and older societies) require ongoing responses to meet present and future global healthcare needs [1]. Health professions are already struggling to fulfill their mission of comforting, curing, and caring for people in need [2]. Since 2000, three seminal reports have indicated that worldwide health-professional education must equip clinicians for the changing needs both of patients and of healthcare systems. Core competencies put forward by the World Health Organization (WHO), (2005) for all health professions include patient-centeredness, partnering with patients, providers and communities, quality improvement, the use of information and communication technology, and a public health perspective of care. Based on demographic and societal developments and population needs, in ‘the future of nursing: leading change, advancing health’, the Institute of Medicine (IOM) recommends that nursing education focus7 on older people, emphasize collaboration and adopt a patient- and family-centered perspective. Further, the authors recommend re-directing nurse education towards primary care settings, switching its center to community care and prevention rather than acute care. The groundbreaking “Health professionals for a new century: transforming education to strengthen health systems in an interdependent world” report suggests that using transformative learning as instructional reform will lead to more equitable, more efficient health systems [3]. In sum, nurses should be educated to deliver patient and family-centered care as members of inter-professional teams embedded in the community, emphasizing evidence-based practice, quality improvement approaches, and full use of information technology [1, 3, 4]. In developed and developing countries alike, a healthcare focus is moving towards people living with non-communicable chronic conditions, e.g., heart disease, diabetes and dementia, and the need to care effectively for these groups and their families via inter-professional collaboration [1]. Calling for enhanced clinical skills and an expanded scope of practice for all health professionals, these new complexities are reflected in five basic competencies: 1) patient-centered care; 2) partnering; 3) quality improvement; 4) information and communication technology; and 5) a public health perspective.

This expansion does not invalidate existing competencies, e.g., evidence-based practice and ethical care; rather, it underscores the need for new ones to complement them. And while they apply to *all* health professionals, these competencies are particularly crucial for nurses, whose duties span health system levels and settings from remote primary care clinics to urban acute care hospitals [1].

With their advanced clinical skill sets and broad scope of practice, Advanced Practice Nurses (APNs) strengthen healthcare systems by providing expert care, especially to people who are older and/or have chronic diseases [5–7]. The International Council of Nurses defines an Advanced Practice Nurse (APN) as a registered nurse who holds a master’s degree and “has acquired the expert knowledge base, complex decision making skills and clinical competencies for expanded practice, the characteristics of which are shaped by the context and/or country in which s/he is credentialed to practice” [8]. APN competencies incorporate direct clinical care (e.g., clinical assessment, clinical interventions, advanced health assessment skills, decision making and diagnostic reasoning skills, case management). They also include expert coaching and guidance (communication, facilitation, reflection and coaching skills), consultation (patient education), research skills (translational research, evaluation of healthcare services), clinical and professional leadership (practice development, planning, implementation and evaluation of programs, change management, quality management), collaboration (intra- and interprofessional), and ethical decision-making skills [9, 10]. To equip nurses for their new responsibilities and ensure a well-educated health workforce, ‘nurse educators need to keep up with a rapidly changing knowledge base and new technologies’; ‘Nursing education, in addition to conveying necessary skill sets, needs to provide students with the ability to mature as professionals and to continue learning throughout their careers’ [2].

### Nursing education in Europe in the wake of the Bologna process

With the Bologna Declaration; the European Union’s ministries of education collaborated to develop a comparable, compatible and coherent system for European higher education [11]. The ultimate goal is a system of academic degrees that are easily recognizable and comparable (i.e., bachelor, master, PhD), promote mobility among students, teachers and researchers, and ensure high-quality learning and teaching. Key focus areas include lifelong learning, employability, funding, degree structures, and international openness, as well as data collection and quality assurance employing the European Credit Transfer System (ECTS). ECTS credits are numerical values that express student time investment, with one credit mirroring 25–30 h of student work in- and outside the classroom.

For nursing education, the Bologna Declaration has led to numerous developments to harmonize Europe’s diverse academic structures and regulations. After a transition of several years, professional nursing education is now offered in bachelor, master’s and doctoral programs recognized across the EU [12]. In Switzerland, a non-EU country, the Federal Council ranked implementation of the Bologna reforms as essential to its higher education system. Also within the Bologna process framework, the Swiss University Conference published directives for the coordinated renewal of teaching at Swiss Universities in 2004 [13]. Switzerland was one of the last European countries to adopt academic nursing education into its higher education system [14].

### The Swiss health care system and nursing education

The Swiss health care system serves a population of eight million inhabitants. Switzerland is a federation of 26 Cantons (States) and its healthcare system determined by the country’s political federalist system, characterized by a decentralized 3-level structure – federal, cantonal, and municipal - and a high degree of local autonomy. The main responsibilities at the federal level include legislative and supervisory roles e.g. the regulation of the mandatory health insurance coverage; the promotion of science, research and university based health professional education (e.g. physicians); and the education of non-university-based health professions including nurses. The cantons are in charge of the provision of health care and partial funding health care providers such as hospitals and education of health care professionals, as well as the implementation of federal laws. Finally, the cantons and its municipalities guarantee health delivery to the population including ambulatory and home health care, acute, specialty and long-term care with its institutions such as general practices, hospitals and nursing homes among others [15].

Nursing education in Switzerland has undergone major reforms and changes over recent decades. Until the early 1990s, Swiss nursing curricula lasted 3 years with three options i.e. general adult nursing, mother and child nursing and psychiatric nursing, offered in more than 150 - often hospital-based- nursing schools across the country.

In the Swiss educational system professional nursing professional education is embedded in the upper secondary and tertiary levels. Upper secondary level education includes vocational and education training programs and tertiary level education includes a) higher education programs at universities and Universities of Applied Sciences (i.e. BScN), and b) professional education and training programs at colleges (i.e. Diploma). Career pathways for professional nurses include many professional roles in a variety of clinical and non-clinical or mixed settings and arrangements. In the last 20 years, new and more clinically focused roles for well-educated nurses have evolved as career paths in the last including clinical nurse specialists and ANPs [16–19]. For instance, University hospitals implemented clinical career ladders, to develop and establish ANP roles requesting that “ANPs are holding a Master’s degree” [20].

### Academic nursing education at the University of Basel

In 2000, the newly launched Institute of Nursing Science (INS) at the University of Basel’s Faculty of Medicine was the first academic nursing institute at a Swiss university. As a pioneering organization, the Institute’s mission statement emphasized strengthening nursing practice and improving clinical outcomes through education, research and skill development within an interprofessional healthcare context, with the goal of its MScN program to prepare advanced nursing professionals. At that time, Advanced Nursing Practice (ANP) was a radically new concept for Switzerland, as graduate nursing education had previously focused on preparing nurse scientists, educators and managers for the German speaking world. Guided by the health care needs of the Swiss population, the program’s strong focus was on self-management (by people living with chronic illnesses), patient safety and quality, and new models of care. From 2000 to 2013, the MScN curriculum was a 3-year full-time program combining a transitional BSc with a consecutive MSc program with part-time options. The MScN incorporated three modules: 1) ANP; 2) research; and 3) leadership and collaboration–each involving several full-semester courses (*see* Table 1). Beginning with 28 students in 2000, the INS has become a key player in higher nursing education, research and clinical practice development across German speaking Europe [14]. Although not fully congruent with the Bologna regulations, the University of Basel supported the INS’s 60-credit-point ECTS transitional BScN study program. After the first years, however, this exceptional permission was debated and INS was invited to present ideas for the program’s transformation into Bologna conformity. On the occasion of this request, INS decided for a rigorous and prudent approach with the overall aim to strengthen and update the original curriculum to meet the need for academically trained nurses as the vanguard of a 21^st^ century’s healthcare system.

**Table 1:** Curriculum of the INS (modules and courses) 2000–2013

Research	Advanced Nursing Practice (ANP)	Leadership & Collaboration
3rd year – MScN 2 (Focus: Community/Society) (plus elective courses)		
• Master Thesis	• Public Health	• Master Thesis
-“Quantitative” seminar	-Epidemiology	-“Project” Seminars
-“Qualitative” seminar	-Health politics	
	-Health economy	
	• Nursing goes public	
	-Media project	
2nd year – MScN 1 (Focus: Family/Community) (plus elective courses)		
• Statistics III	• Community assessment	• Leadership
• Research methods II:	• Patient safety & Quality	-Action Learning
-Qualitative methods	• Clinical Assessment II	-Evidence based practice (EBP)
• Proposal writing	• Pharmacology	-Project management
	• Genetics	-Interdisciplinary collaboration
	• Family care	
1st year – Bachelor transition program (Focus: Individual)		
• Statistics I & II	• Pathophysiology	
• Scientific writing	• Clinical Assessment I	
• Research methods I	• Role of APN /Work shadowing	
-Quantitative research methods	• Communication skills I	
-Qualitative research methods	• Living with chronic Illness	

## Methods – the work packages

Prior to the curriculum revision, we formulated three central aims: 1) to review relevant reports in view of local and international health professional education; 2) to analyze the content of the curriculum’s semester courses via the analytical insights of reports, combined with alumni perceptions of professional gaps; and 3) to re-design and/or overhaul the courses as appropriate.

**Fig. 1:**
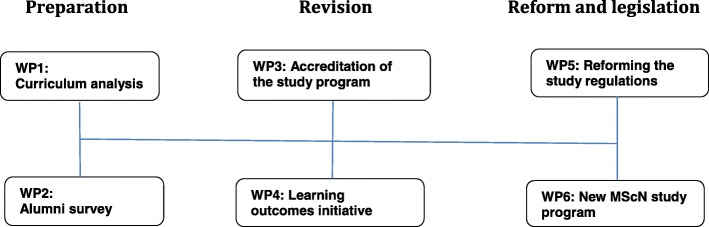
Study reform process work packages

The reform process consisted of three interrelated phases–*preparation*, *revision* and *reform and legislation*–including six thematic work packages (WPs). For the *preparation* phase, i) relevant reports were analyzed regarding health professional education, followed by ii) a curriculum analysis using insights from publications identifying new content requirements (WP 1). A structured survey helped explore alumni experiences and opinions about the program, and highlighted perceived professional gaps (WP 2). The *revision* phase included the MScN program accreditation process (WP 3) and the reformatting of all courses, as required by the Bologna Declaration, to focus on learning outcomes, i.e., to competencies, rather than pure knowledge (WP 4). The *reform and legislation* phase included study regulations adapted to fit the University of Basel’s legal requirements (WP 5) and the newly designed MScN curriculum, with its core study program and two study track possibilities, *Research* or *ANP* (WP 6). The entire study program reform process was led by a curriculum revision team (CuRT) consisting of INS faculty and administrative support, as well as external consultancy from higher education experts on process reflection and feedback. The CuRT devised the action plan that guided and coordinated the six work packages’ implementation (see Fig. 1).

### Preparation phase

#### WP 1 - curriculum analysis

For the curriculum analysis, the CuRT used a self-developed analysis matrix to systematically review each semester course, paying close attention to the WHO’s five core competencies [1] and Hamric’s APN competencies [9] as evaluation criteria (Table 2). To determine whether individual courses addressed the postulated competencies for each of these criteria, we used a simple rating scale: 0 = not addressed; 1 = partially addressed; 2 = mainly/fully addressed; or not applicable.

**Table 2:** WHO core competencies for caring for patients with chronic conditions & APN competencies

1. Patient-centered care	
-Interviewing and communicating effectively	
-Assisting changes in health-related behaviors	
-Supporting self-management	
-Using a proactive approach	
2. Partnering	
-Partnering with patients	
-Partnering with other providers	
-Partnering with communities	
3. Quality improvement	
-Measuring care delivery and outcomes	
-Learning and adapting to change	
-Translating evidence into practice	
4. Information & communication technology	
-Designing and using patient registries	
-Using computer technologies	
-Communicating with partners	
5. Public health perspective	
-Providing population-based care	
-Systems thinking	
-Working across the care continuum	
-Working in primary health care-led systems	
Advanced Practice Nurses competencies	
-Advanced direct clinical care	
-Expert coaching and guidance	
-Consultation	
-Collaboration	
-Clinical and professional leadership	
-Ethical decision-making skills	
-Research skills	

The CuRT analysed the curriculum and reviewed the courses. By consensus, each of the courses was rated to indicate its fit with the WHO core competencies and APN competencies. Reviewing and analyzing the curriculum’s 25 courses (10 from the BScN transition program and 15 from the MScN study program) resulted in an overview indicating whether applicable WHO core competencies and APN competencies were addressed, partially addressed or not addressed. Our expert group’s findings indicated that the majority of courses’ content matched the criteria. First, where applicable, the five WHO core competencies were addressed in all courses at least to some extent (specific competencies were not applicable in certain research or ANP courses). Second, APN competencies were addressed partially or fully in all courses in all areas of interest.

Based on these findings, and considering recent publications on nursing education [3] and future directions of nursing [2, 4], INS faculty discussed priorities for the curriculum revision. Based on lecturer experience in previous years, we also considered integrating collaborative, non-formal evaluations of student outcomes vs. program aims and faculty expectations. More specifically, the faculty reached consensus on courses in need of re-design or new development in view of transformational learning, use of technology, collaborative care and emerging nursing issues. As this is an extremely context-oriented process, many decisions were made according to the specific healthcare needs of the Swiss population or innovations in the Swiss educational landscape. During the same process, it was also decided to further elaborate a series of topics for future courses.

- Advancing clinical education (e.g., small-group tutorials and individual internships)
- APN role development to strengthen implementation of new models of care
- Advanced research methods to strengthen competencies in quantitative and qualitative approaches
- Philosophy of science to provide basics of epistemology and schools of thought
- E-Health to provide basic understanding of an emerging field that is transforming healthcare
- Genomics as an introduction to another transformative healthcare field, e.g. personalized medicine

Further, it was decided to introduce e-learning and blended learning methods in selected courses as appropriate and to consider inter-professional education, e.g., including medical students (in collaboration with their departments within the Medical School). All of the above-mentioned topics were then further elaborated and delegated to INS lecturers and CuRT for stepwise development and implementation (see WPs 5 and 6).

#### WP 2 - alumni survey

In developing the MScN study program and APN roles in Switzerland, it was extremely important to explore our graduates’ perspectives on their professional roles and clinical positions, as well as their experiences with the INS study program and their suggestions and visions regarding future needs. Therefore, in December 2008, all 76 alumni were contacted via e-mail and invited to participate in the first INS alumni survey. The survey was conducted via a self-developed paper-and-pencil questionnaire with standardized response categories to tick and free text comment boxes where appropriate. Its domains included socio-demographic and professional status information, evaluation of the study program, APN role status and future prospects for APN development.

Of the 76 alumni (90% female) initially contacted, 51 completed the questionnaire, yielding a response rate of 67%. The mean age of participants was 38 (range: 28–52) years at the time of graduation. Two-thirds (65%) worked in hospitals, 24% in educational institutions and the remaining 13% in long-term, community or mental health care settings or other positions. All were employed either full- or part-time. Although a clear majority (57%) of respondents worked in APN or similar roles, many expressed regret that their current roles did not allow the application or expansion of clinical competencies, or that they felt insecure taking clinical decisions autonomously. Results confirmed that only a minority of respondents has integrated Hamric’s suggested APN core competencies, especially clinical skills in their field of expertise, in their care practices [9]. Involvement in direct clinical patient care and support of care teams in ethical decision-making and other activities received the highest and lowest values–respectively 24 and 5%.

The INS faculty team’s discussion of the survey findings additionally inspired curriculum revision activities, particularly regarding new topics or changes elements of existing courses, e.g., methods and didactics. More specifically, considering the pioneering character of such roles in the Swiss healthcare context, these results highlighted the need for more advanced clinical and scientific training in practice settings, as well as increased support in terms of APN role development.

### Revision phase

#### WP 3 - accreditation of the master study program

After several years of delivering a pioneering MScN study program, the INS faculty arranged an independent review by an official agency. In December 2008, the INS requested national accreditation by the Swiss Agency for Accreditation and Quality Assurance (AAQ) (https://aaq.ch/). After an initial meeting between representatives of the INS and the agency, the schedule for the accreditation process (a self-evaluation report, an expert onsite visit, and an expert report with recommendations) was discussed and confirmed. Following the agency’s standard procedure, the necessary application documents were drafted and submitted, after which the experts’ 2-day site audit took place in May 2009. The agency forwarded the official report and recommendations to the Swiss University Conference, which granted the revised MScN study program unconditional accreditation for 7 years.

As standard accreditation procedure, the expert group also proposed a set of recommendations for strengthening the study program, e.g., *“Examine the ways in which greater use could be made of IT in terms of supporting the learning environment, providing some ‘virtual mobility’ (especially for those whose options for ‘physical’ mobility are limited by work, family and other (non-study) commitments.”* While these recommendations were not conditions of the accreditation itself, they were congruent with IOM recommendations [4], and thus were integrated into planned didactic changes by promoting blended-learning course formats. Thus, recommendations by AAQ were, first, adopted directly in the new MScN study program (WP 6) and, second, incorporated into the action plan for the current strategic planning phase (2014–2018): course re-design demands critical review of IT and e-learning options at the institutional / educational management system level, as well as at the faculty level, requiring additional preparation for both lecturers and students [3].

#### WP 4 the learning outcomes initiative

Alongside the transformation of traditional (i.e., knowledge-based) descriptions of qualifications and qualification structures into competency-based aims and objectives, the Bologna process stipulates the definition of learning outcomes for all modules and programs (i.e., bachelor, master, PhD) in tertiary institutions [21]. Learning outcomes are used to express which competencies learners will be expected to achieve and how they will be expected to demonstrate that achievement at the end of a learning activity. Although, structurally, the INS’s master study program was implemented according to Bologna from the beginning, at the level of individual semester modules and courses, its instructional mode still followed a traditional teacher-centered methodology.

The new learning outcome-oriented perspective’s potential to shift the INS from a teacher-centered to a student-centered approach was well received by both faculty and CuRT. Therefore, via a small teaching-the-teachers (*ttt*) project group, consisting of three curious, highly motivated and experienced lecturers, CuRT prepared the learning outcomes initiative. Delivered in 2010 and audited collegially by the University of Leuven, Belgium’s curriculum design department, this *ttt* project laid the groundwork not only for a discussion and subsequent accord on how best to switch to the new model, but also for ongoing contact and mutually beneficial curriculum design consultancy and educational expert exchanges.

### Reform and legislation phase

#### WP 5 reforming the study regulations

The overhaul of the INS MScN study program regulations was an intense process, requiring close collaboration between university administrative experts, faculty, pedagogical authorities and legal experts. From its beginning in 2009, when the INS submitted a formal request to the University’s board of education, a tenacious INS CuRT core group navigated the process through the course of its development, including a host of formal and legal issues, to its official release, in 2013, by the University Council.

As early direct results of the curriculum reform, the existing accelerated BScN program regulations were terminated, and the MScN program regulations underwent major revisions. Finally, the University Council’s decision to implement the revised study regulations as of October 2013 allowed the new MScN program’s first student cohort to commence in the 2014 fall semester.

#### WP 6 the new MScN study program

**Fig. 2:**
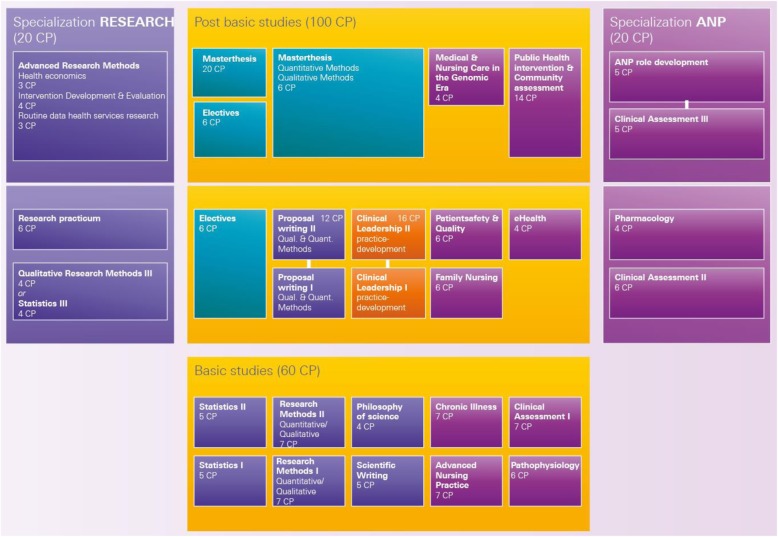
Curriculum of the new Master of Science in Nursing study program

The new dual-track MScN program allows students to choose between two tracks: *Research*, leading to academia or other science-related domains; and *ANP,* leading to advanced clinical practice. In either case, the study program is composed of a 1-year basic study program of 10 single-semester courses (60 ECTS) and a 2-year core study program with a total of 12 single-semester courses, including electives and 4 special courses for each of the chosen tracks (see Fig. 2). Entry criteria for the MScN study program include either a BSc in Nursing or Midwifery plus two years of professional experience, or a nursing or midwifery diploma from a tertiary-level degree program, a successful matriculation exam (e.g., Matura, Abitur) and two years of full-time experience in a healthcare profession. Course dispensations are possible and vary depending on the candidates’ educational backgrounds.

The 2010 IOM recommendations for nursing education [4], Frenk et al. [3], the results of the alumni survey and our MScN curriculum analysis all facilitated major changes to many of our courses. To strengthen research and clinical education, we decided on two actions. First, we would redesign first year courses; and, second, we would create two specializations–‘research’ and ‘APN’–as study options for the post-basic study program (20 KP ECTS each, see the right and left sides of Fig. 2). To enhance research education, we redesigned first-year research courses (i.e., *Research I, Introduction to quantitative and qualitative methods*, and *Research II, Expansion of quantitative and qualitative methods*). To broaden the program’s ethical foundation, we added one course in Philosophy of Science. The following second- and third-year courses were newly developed or redesigned: *Qualitative research (specific qualitative methods)*, *Advanced Research Methods* (comprised of 3 courses: *Health economics*, *Using large routine datasets for health services research*, and *Intervention development and evaluation*). These course revisions necessitated major changes to a mandatory research internship (whereby students join existing research groups and experience ‘research in practice’). To extend clinical education, the following second and third year courses were newly established or redesigned: *Clinical Assessment III* (individual clinical internships mentored by physicians/APNs in their field of expertise); and to introduce ANP roles, *APN role development: working through the PEPPA (Participatory, Evidence-based, Patient-centered Process for APN role development, implementation and evaluation) framework* [22].

To adapt to the latest developments in terms of nursing knowledge, we tailored our educational offerings to fit local conditions and integrate new technology [2, 3]. Therefore, two two pilot courses–*eHealth (Advancing the use of information technology in healthcare*) and *Genomics (Medical and nursing care in the genomic era)*–were included in the core study program. The comparison of existing content with IOM-issued core competencies for nurses [2] further revealed the need for a stronger emphasis on the public health perspective in intervention development; as a result, *Public health and community care (community assessment and interventions)* was redesigned. To exemplify the results of the curriculum and course development syllabi with general objectives, Table 3 displays the learning outcomes and content of two new courses.

**Table 3:** Examples of course objectives, learning outcomes and content

APN role development (ECTS: 4)	
General objectives: Following the ‘Participatory, Evidence-based, Patient-centred Process for APN role development, implementation and evaluation’ (PEPPA) framework, based on an unmet healthcare need across the care continuum, students develop a model of care for a specific patient population. In close collaboration with key stakeholders (e.g., other medical professions, patients and families, external support system representatives), they further develop a model of care, meeting the care need and define their new, expanded role within this new model of care.	
Learning outcomes (selection): On completion of the module students will be able to: a) define a patient population and identify an unmet health care need; b) identify and gain the views/opinion of stakeholders regarding the unmet health care need and a potential ANP role; c) engage stakeholders in the development and implementation of the proposed model of care and ANP role; and d) develop and present an overview of the proposed model of care and ANP role using a logic model	
Content (selection): Action learning, role development principles, PEPPA framework / toolkit	
Student evaluation: Each student will work through the PEPPA model steps and hand in a completed needs assessment, stakeholder analysis, logic model and a summary reflection on own experiences with applying the PEPPA framework.	
Using large routine datasets (LRD) for health services research (ECTS: 3)	
General objectives: To enhance the in-depth understanding of the planning and implementation of the analysis of large routine data in the context of health services research. Today’s healthcare systems provide a wide range of data sources like large discharge datasets, epidemiological registries or data from the electronic health record, which offer many opportunities for health and nursing research. The course will provide students the basis to plan and conduct LRD analyses in the context of their own area of research.	
Learning outcomes: On completion of the course, students will a) understand the basic steps in the analytical process of LRD sets; b) develop and assess answerable research questions in the context of LRD; c) evaluate scope and limitations of popular analytical techniques in the context of LRD; d) understand and apply principles of reproducible research and e) plan, conduct and present a contained LRD project	
Content (selection): The complete process from importing, preparing, analyzing, reporting and presenting the data will be covered; unique design features to be considered in the planning and stages of a study so that specific methods can be employed during the analysis.	
Student evaluation: Written analysis plan and written report	

With the 2014 fall semester the new MScN study program was in place. I.e., from that point on, students who enrolled automatically started in the new program, while those who started earlier could either transfer to the new program or complete their studies in the old one, which fading out completely by 2016. After the basic study program, students are asked to choose one of the study tracks. Whichever specialization they choose, they have the option to change to the other, but will still be required to meet all of that track’s course requirements. Although most of our students initially choose the ANP specialization, enrollment is now shifting slightly toward research. Given this shift, and the growing proportion of master study-based research papers published in recent years, we are optimistic that interest in research-driven careers is increasing.

## Discussion and conclusions

From the early days of Switzerland’s first MScN program in 2000 to the enrolment of the first cohort to study under the new curriculum in 2014 in this profoundly altered educational context, the overhaul of this program’s curriculum facilitates our mission to educate and encourage nurses not only to take up leading roles, but “to leverage opportunities to improve frontline care. We develop, promote and lead the implementation of research driven innovations through clinical partnerships, and drive innovation in Advanced Nursing Practice education in the German speaking world’ (https://nursing.unibas.ch/de/ins/leitbild/). The INS’s rigorous approach, with a sharp focus on the Swiss population’s current and future care needs, can serve as a framework for others revising a nursing curriculum at the MScN level.

Our method of comparing current curriculum content with WHO core competencies and ANP competencies was generally successful. Regarding overall content, our educational direction was well-corroborated; and from the program’s implementation, the curriculum was well aligned with APN competencies and with most of the WHO’s 5 proposed core healthcare competencies [1]. We realized that technology was well represented in course content, but needed strengthening regarding instructional design. Moreover, supporting Frenk et al. (2010), seminal IOM reports (2011) pointed out the importance of balancing strong clinical and research options for advanced nurses. Reflected in the core findings of our alumni survey, these reports led the way to our twin (‘Research’ and ‘ANP’) specializations. Frenk et al. (2010), for instance, stressed that the target outcome of instructional reforms should be transformative learning based on the development of professional competencies adapted both to fit local contexts and to promote inter-professional learning. Our *ANP role development* course integrates these elements; it is conceptualized in terms of clinical activity across two semesters, during which the students develop their future ANP roles in their local contexts and in collaboration with stakeholders, e.g., physicians and other professionals. Experiences with two student cohorts already indicate that, especially in Switzerland, this course facilitates transition to novel, advanced professional roles (IOM, 2011).

Regarding individual courses, the structured curriculum analysis indicated how fully each course addressed the requisite ANP and WHO professional competencies. The interpretation of the results posed various challenges, as from the commencement of the revision process it was never clearly confirmed whether all criteria needed to be applicable to all courses. For example, the “Patient centered care” criterion would not apply to the research module, or to courses on scientific method or statistics but rather to courses of *Clinical Assessment* or *APN role development*. Therefore, to overcome these and other concerns regarding the rationale for specific course content adaptations, we addressed the applicability of the core competency criteria pragmatically and focused on specific courses. To provide essential information on which courses required changes regarding the ANP and Research specialization, any concerns were dealt with via discussion and consensus within the CuRT and faculty. A clear definition of the context in which each revision is being made, i.e., regarding specific population needs or the coverage of educational offers by other schools, may enhance the validity of decisions regarding new courses or course redesign.

Following decisions on which elements to update or replace, the creative and challenging endeavor of structuring the material was conducted by the CuRT. They worked through each issue by asking basic questions, trying ideas, puzzling, thinking aloud, and often laughing. This team approach to problem solving, which nurtured unconventional thinking and questioning apparently self-evident constructs, often became the initial step for groundbreaking changes and innovative solutions. For example, by asking whether all students actually benefit by doing *Clinical Assessment 2*, a problem of allocating ECTS credits was resolved by integrating that course into the ANP track; i.e., recognizing that advanced clinical assessment actually has little relevance to students in the Research track, it was allocated to the ANP specialization. After successfully framing the new master’s program, study plans were necessary for students in both tracks.

Characterizing learning outcomes as target competencies according to “Bologna” was a vital change; and the precept that curricula need to be competence-driven has remained valid throughout our journey. With the long-range goal of evaluating and ensuring quality care outcomes for the population, the students’ acquisition of advanced competencies via the integration of competency-driven instructional design was at the core of both the IOM recommendations (2011) and Frenk et al. (2010). All INS course leaders and lecturers engaged in drafting, reviewing and finalizing the courses of the MScN curriculum. This level of individual investment promoted identification with the new approach, providing a strong basis for its sustainability.

Study regulation reform demanded intense concerted collaboration between the members of a committee including CuRT, representatives of the University’s student administration, the legal department, and the rector’s office. This group met every 1–2 months over a period of 18 months. Along with the rector’s office’s provision of a project plan, including milestones, the University’s contributions of services (e.g., meeting rooms) as well as the sincerity of the collaboration facilitated a lean and unhindered process from start to finish. However, while all participants were eager to contribute and proceed, it was essential to allocate ample time for discussion of meanings or concepts, and to develop a common understanding first of the INS’s fundamental aims, then of the curriculum revision’s ultimate objectives.

This semester, 1 year after launching the new curriculum and its regulations, the INS experienced an unexpected surge of students opting for the ANP track. Apparently, while our students clearly appreciate the practical aspects of our BScN program, few understand the excitement, fulfilment and profound value of nursing research. Obviously, we must work harder to expose students to the many rewards of existing nursing research. In the coming years, by integrating promising students into research groups and offering research internships, more will choose the research track. Additionally, inviting students from other disciplines to join our research-track courses will foster robust inter-professional exchange among the participants.

### Implications and next steps

Recommendations from the above-mentioned reports and results of our analyses not only informed our curriculum redesign but were also considered for the institute’s 2014–2018 strategic planning phase. For education, for instance, four major goals and corresponding action points in the domains relevance, quality and innovation, impact, and sustainability were defined:

1. The INS educational program will prepare nurses with 21st century competencies to address healthcare needs and lead ANP education in German speaking countries (relevance).
2. The INS educational program will reflect international standards in nursing education, including innovative teaching (quality & innovation).
3. INS graduates will be recognized as effective change agents and pacemakers in the Swiss healthcare system (impact).
4. The INS will attract highly talented students and faculty by creating a stimulating and supportive work environment with a strong sense of community (sustainability).

To achieve Goal 1, acknowledging recent changes across the Swiss healthcare landscape, re-thinking professional roles for sustainable and equitable healthcare will entail ongoing curriculum review; i.e., considering the IOM recommendation to increase education of Nurse Practitioners for primary care roles. For Goal 2, the integration of technological elements into course content was followed; however, implementation of technology needs to be increased regarding instructional design. Goal 3 refers to our graduates’ future professional roles and impacts: very positive feedback from diverse clinical settings indicate that the clinically located and supervised *Clinical Assessment III* and *ANP Role Development* courses are already increasing the number of INS graduates in advanced clinical roles as well, while building mutual trust and respect through interprofessional collaboration. For Goal 4, it can be hypothesized that the increasing numbers of applicants reflect the attractiveness of our revised study program; however, this hypothesis needs to be substantiated via empirical methods. Finally, the University of Basel’s Institute of Nursing Science remains committed to integrating international developments and recommendations into its strategic planning, and to providing ongoing innovative high quality MScN education in Switzerland.

## Abbreviations

ANP: Advanced Nursing Practice
APN: Advanced Practice Nurses
BScN: Bachelor of Science in Nursing
CuRT: Curriculum revision team
ECTS: European credit transfer system
EU: European Union
INS: Institute of Nursing Science
IOM: Institute of Medicine
MScN: Master of Science in Nursing
WHO: World Health Organization
WP: Work packages
